# Evidence of Oligoclonal Bands Does Not Exclude Non-Inflammatory Neurological Diseases

**DOI:** 10.3390/diagnostics11010037

**Published:** 2020-12-28

**Authors:** Katharina Pannewitz-Makaj, Ulrich Wurster, Konstantin Fritz Jendretzky, Stefan Gingele, Kurt-Wolfram Sühs, Martin Stangel, Thomas Skripuletz, Philipp Schwenkenbecher

**Affiliations:** Clinical Neuroimmunology and Neurochemistry, Department of Neurology, Hannover Medical School, 30625 Hannover, Germany; Pannewitz-Makaj.Katharina@mh-hannover.de (K.P.-M.); wurster.ulrich@mh-hannover.de (U.W.); Jendretzky.konstantin@mh-hannover.de (K.F.J.); gingele.stefan@mh-hannover.de (S.G.); Suehs.Kurt-Wolfram@mh-hannover.de (K.-W.S.); stangel.martin@mh-hannover.de (M.S.); skripuletz.thomas@mh-hannover.de (T.S.)

**Keywords:** oligoclonal bands, cerebrospinal fluid, intrathecal immunoglobulin production, Reiber’s diagram, biomarker

## Abstract

Cerebrospinal fluid analysis is an essential part of the diagnostic workup in various neurological disorders. Evidence of an intrathecal immunoglobulin synthesis, as demonstrated by Reiber’s diagram or the more sensitive oligoclonal bands (OCB), are typical for neuroinflammatory diseases, and normally not expected in non-inflammatory neurological diseases. Therefore, patients with non-inflammatory neurological diseases are often used in control groups in studies investigating autoimmune diseases of the central nervous system. However, data about the frequency of intrathecal immunoglobulin synthesis in non-inflammatory neurological disease are scarce. The cerebrospinal fluid (CSF) records of a total of 3622 patients were screened and 2114 patients included with presumably non-inflammatory neurological diseases like dementia, idiopathic peripheral neuropathy, motoneuron disease, stroke, and epileptic seizures. Evidence of an intrathecal immunoglobulin synthesis can be found with low frequency also in non-inflammatory neurological diseases. A much higher rate of patients showed intrathecal immunoglobulin synthesis as demonstrated by OCB than by Reiber’s diagram. In patients with disorders of the peripheral nervous system the frequency of OCB was much lower than in patients presenting with central nervous system manifestations. Evidence of an intrathecal immunoglobulin synthesis should not automatically lead to exclusion of non-inflammatory neurological diseases but should rather prompt the way to investigate for the origin of the intrathecal immunoglobulin synthesis.

## 1. Introduction

The analysis of cerebrospinal fluid (CSF) is one of the most important laboratory methods in the diagnosis of a broad spectrum of neurological diseases [1,2]. In the 1960s, Löwenthal and colleagues described an abnormal immunoglobulin fraction in the CSF of patients with multiple sclerosis by using electrophoresis [2,3]. The detection of these electrophoretic patterns called oligoclonal bands (OCB) in the CSF but not in the corresponding serum is indicative for the presence of IgG-secreting clones within the central nervous system (CNS) [4,5]. CSF-restricted OCB are frequently found in various inflammatory CNS diseases and attain almost 100% in multiple sclerosis [6,7]. The diagnostic significance of OCB as a biomarker has been emphasized by the implementation in the latest revision of the McDonald criteria for multiple sclerosis as a substitute for dissemination in time [8].

Another method to detect an immunoglobulin synthesis within the CNS has been developed by Reiber and colleagues that is now called Reiber’s diagram [9]. It is a quantitative method, which can also demonstrate an intrathecal production of immunoglobulin A and M, but is less sensitive than OCB [10,11,12]. CSF investigation including determination of OCB and Reiber’s diagram is crucial, not only when inflammatory neurological diseases are expected, but also to exclude differential diagnoses in non-inflammatory neurological diseases [2]. Although both methods, OCB and Reiber’s diagram, have been well-established over decades in the diagnostic work-up, data about the frequency of intrathecally produced immunoglobulins in non-inflammatory neurological disease are scarce. We therefore aimed to evaluate the frequency of an intrathecal immunoglobulin production detected by OCB and Reiber’s diagram in non-inflammatory neurological diseases.

## 2. Materials and Methods

### 2.1. Patients

For this retrospective study CSF data of all patients who received a lumbar puncture at the Department of Neurology of the Hannover Medical School in the time from 2013 to 2015 were screened for non-inflammatory neurological diseases. A total of CSF data from 3622 patient were investigated. Laboratory testing (antinuclear antibodies, anti-DNA antibodies, antiphospholipid antibodies, antineutrophil cytoplasmic antibodies, autoimmune encephalitis antibodies, paraneoplastic antibodies, HIV, and antibodies against Borrelia burgdorferi, and treponema pallidum), and magnetic resonance imaging were performed to exclude autoimmune causes such as multiple sclerosis, neuromyelitis optica spectrum disorders, connective tissue diseases, vasculitis, or infectious diseases, whenever these diseases were suspected. Demographic and clinical data were extracted from medical records and evaluated. Patients were only included when CSF cell count was within normal range (<5/µL). Patients were then assigned to the following groups: (1) symptoms without a neurological deficit (e.g., headache), (2) peripheral neuropathy, (3) neurovascular disease, (4) epileptic seizure, (5) encephalopathy and delirium, (6) muscular disease, and (7) cerebrospinal fluid flow disorders.

CSF examination was performed in patients with neurovascular disease to exclude inflammatory vascular disorders like autoimmune vasculitis or parainfectious vasculitis or to exclude encephalitis due to consciousness disturbance. In patients with a first epileptic seizure, CSF diagnostics are part of routine work-up. Autoimmune encephalitis antibodies were determined in these patients in cases of subacute onset of working memory deficits, altered mental status or psychiatric symptoms and were negative for all patients.

### 2.2. CSF and Serum Analytical Procedures

**Figure 1:**
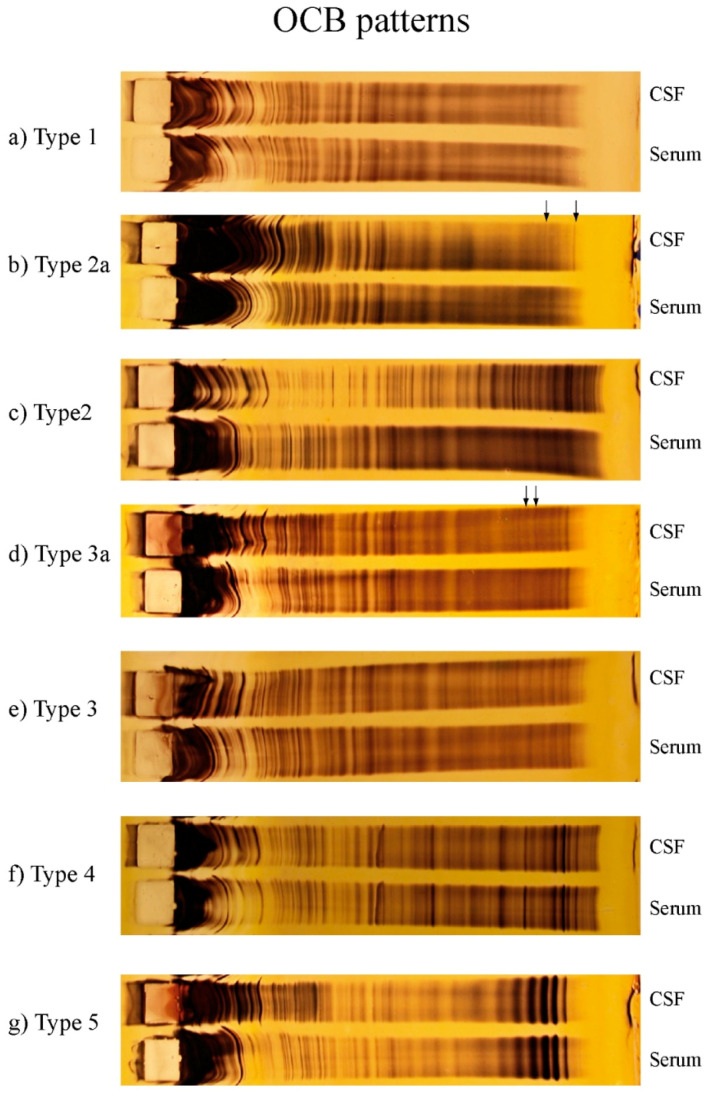
Silver staining after isoelectric focusing on polyacrylamide gel of serum and cerebrospinal fluid (CSF) of oligoclonal bands (OCB) type 1 (**a**), OCB type 2a (**b**), OCB type 2 (**c**), OCB type 3a (**d**), OCB type 3 (**e**), OCB type 4 (**f**), OCB type 5 (**g**). Arrow indicates CSF restricted OCB in borderline types.

Laboratory analyses of paired CSF and serum samples were performed in the Neurochemistry Laboratory of the Department of Neurology of Hannover Medical School as part of the standard diagnostic procedure [13]. CSF cells were counted manually with a Fuchs-Rosenthal chamber. A CSF cell count < 5 cells/µl was defined as normal. CSF total protein was determined by the Bradford dye-binding method, using 500 mg/L as a cut-off. The concentrations of IgG, IgA, IgM, and albumin in the CSF and the corresponding serum sample were measured by latex enhanced kinetic nephelometry (Beckman Coulter IMMAGE, Brea, CA, USA), and the CSF/serum ratios of IgG, IgA, IgM, and albumin were calculated. The CSF/serum albumin ratio, an indicator of the function of the blood-CSF-barrier, was determined by the age-adjusted formula QAlb = 4 + (age in years/15) [1]. For the detection of an intrathecal synthesis, IgG, IgA, and IgM ratios were plotted against albumin ratios according to the method of Reiber [1]. Isoelectric focusing in polyacrylamide gels with consecutive silver staining was used to detect OCB. CSF and corresponding serum sample were adjusted to equal IgG concentrations (20 mg/L). Interpretation of OCB was done blinded by an experienced rater (UW). Following the recommendations of the first European consensus on CSF analysis in multiple sclerosis, five different patterns of OCB were applied [10]. Two or more bands restricted to the CSF were sufficient for a positive rating (type 2 or type 3), but weak type 2 or type 3 patterns with only 2–3 bands were registered separately as type 2a or type 3a. Type 1 and type 4 indicate normal findings (type 1: no evidence for intrathecally produced OCB, type 4: equal numbers of matched bands in CSF and serum, systemic production of oligoclonal IgG). Type 5 shows the presence of a monoclonal gammopathy [6]. Figure 1 depicts typical OCB patterns after silver staining. The methods of the laboratory are validated by the external quality control program of INSTAND [12].

### 2.3. Statistical Analysis

GraphPad Prism version 5.02 was used for statistical analysis. Data are described by means and standard deviation. Statistical significance in categorical data was assessed by Fisher’s exact test and by Chi-squared test. Linear regression was used to calculate age-dependent frequencies. Statistical significance was set for *p*-values to <0.05.

## 3. Results

**Figure 2:**
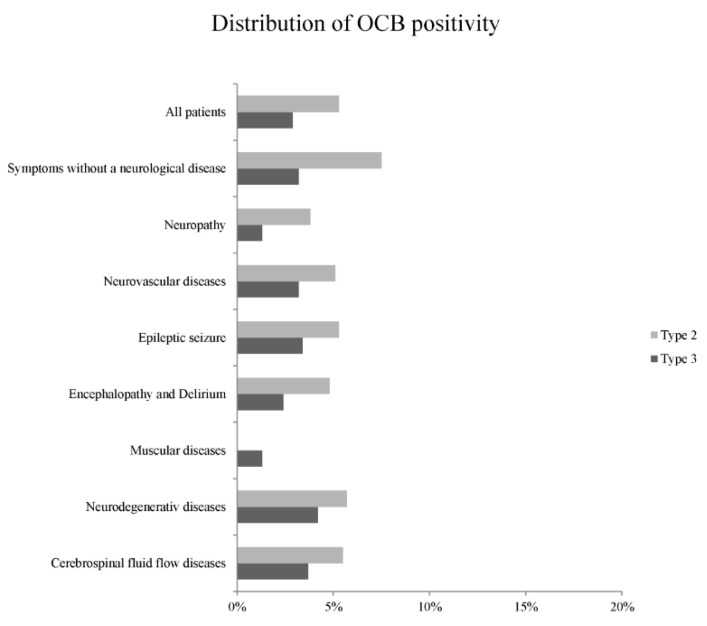
Relative distribution of OCB positivity for all groups investigated. Type 2 compromises type 2 and type 2a. Type 3 compromises type 3 and type 3a.

Table 1 and Table 2, and Figure 2 summarize the frequency and relative distribution of the five OCB types in regard to the clinical manifestations. Since an autoimmune or infectious disease of the CNS was diagnosed or suspected, 1508 patients were excluded after initial screening. A total of 2114 patients with non-inflammatory neurological diseases were then included. When an intrathecal immunoglobulin synthesis was detected by Reiber’s diagram, more than three OCB restricted to CSF were found in all patients (eight patients with OCB type 2 (0.4%) and three patients with OCB type 3 (0.1%)). An isolated IgG synthesis was found in five patients (0.2%) and an isolated IgM synthesis only in one patient (0.05%). The combination of IgG and IgM was detected in four patients (0.2%). An intrathecal IgA synthesis was not detected in any patient.

**Figure 3:**
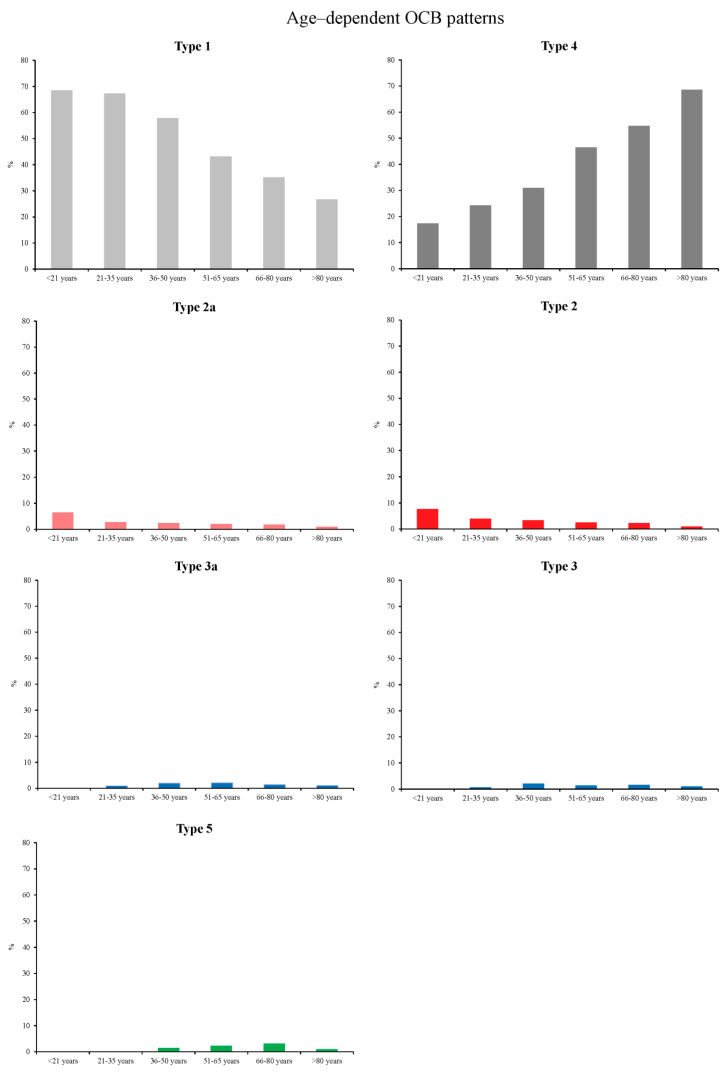
Age–dependent relative distribution of OCB patterns for all patients investigated.

The distribution of age-dependent OCB patterns for all patients is depicted in Figure 3 (4.4% patients < 21 years, 15.2% patients 21–35 years, 19.8% patients 36–50 years, 26.3% patient 51–65 years, 29.4% patients 66–80 years, and 5% patients > 80 years). The frequency of OCB type 1 (no OCB) was significantly decreasing with age (r^2^ = 0.9680; *p* = 0.0004), while the frequency of OCB type 4 (systemic reaction) was significantly increasing (r^2^ = 0.9821; *p* = 0.0001). Furthermore, a significantly declining rate with age was also found for OCB type 2a (r^2^ = 0.7288; *p* = 0.0305) and type 2 (r^2^ = 0.8387; *p* = 0.0103). No statistically significant changes were observed for OCB type 3, 3a and 5.

No significant gender-related differences in OCB patterns could be demonstrated.

Higher percentages of OCB positivity (2–3 and more than 3 OCB restricted to CSF) were identified in patients suffering from a neurological disease with central nervous system manifestations than in patients with peripheral neuropathy or muscular disease. However, these differences were not statistically relevant.

Distinctive OCB positivity (more than 3 OCB restricted to CSF) was most frequently found in patients with cerebrospinal fluid flow disease, in patients with symptoms but without a neurological deficit and in patients with neurodegenerative diseases. The subgroups idiopathic intracranial hypertension and movement disorders showed the highest rate of distinctive OCB positivity. However, differences did not reach a statistically significant level.

Borderline OCB (OCB type 2a and type 3a) were most frequently found in patients with symptoms, but without a neurological deficit, in patients with encephalopathy and delirium and in patients with neurovascular disease. The pain symptom subgroup showed the highest rate of borderline OCB, while no borderline OCB were found in patients with vertigo, trigeminal neuralgia, vestibulopathy, oculomotor palsy, and cerebrospinal fluid leakage syndrome. However, differences between the frequencies of borderline OCB were not statistically relevant. Only in the subgroups trigeminal neuralgia, vestibulopathy and cerebrospinal fluid leakage syndrome no patients with borderline or distinctive OCB positivity were found.

Details of the subgroup neurodegenerative disease are shown in Table 3. No significant difference in frequency of borderline OCB and distinctive OCB positivity could be identified. However, borderline OCB were most frequently found in patients with choreatic movement disorder and distinctive OCB positivity in patients with spinocerebellar syndrome.

## 4. Discussion

CSF analysis including determination of OCB as an indicator for an intrathecal immunoglobulin G synthesis is an integral part of diagnostic work-up, not only when an inflammatory CNS disease is suspected, but also to exclude differential diagnoses in non-inflammatory CNS diseases. The diagnostic value of OCB has been intensively investigated in numerous studies especially in patients with multiple sclerosis [2,6,14,15]. In these studies, control groups usually consist of patients with non-inflammatory neurological diseases. However, these control groups are often composed of different neurological diseases, which apart from being non-inflammatory have nothing in common, such as, for example, motoneuron disease and migraine [16]. Moreover, little is known about the frequency of OCB in these diseases. The heterogeneity of these control groups harbors therefore the risk to misinterpret the significance of OCB in the study group.

In this study we investigated the frequency of an intrathecal immunoglobulin synthesis by determining OCB with isoelectric focusing in polyacrylamide gels and silver staining and calculating intrathecal immunoglobulin synthesis by Reiber’s diagram in one of the largest groups of different non-inflammatory neurological diseases. The main finding is that OCB and to a lesser extent the detection of intrathecal immunoglobulins with Reiber’s diagram can be found in low numbers in all groups of patients with non-inflammatory neurological diseases. A maximum of total (all OCB > 2) OCB positivity was observed for the group of pain (14.6%), followed by movement disorders (12.1%), idiopathic intracranial hypertension (11.9), and headache (11.0%), while oculomotor palsy with 2.4% and muscular disease (1.3%) displayed minimal values. Remarkably, only borderline OCB were detected in the latter two groups with the lowest incidence of total OCB (Table 1). In one of the few comparable studies, in which OCB were investigated in non-infectious neurological diseases, it has been reported that only a small minority of patients with Alzheimer’s disease, cerebrovascular events, vertigo, seizures, amyotrophic lateral sclerosis, and polyneuropathy showed OCB while no OCB were found in patients with Parkinson disease and Bell’s palsy [4]. However, findings of previous studies have to be interpreted with caution due to the low number of patients included. In a recent study by Jesse et al., OCB were found only sporadically in patients with neurodegenerative diseases [17]. In contrast, in another study by Bourahouiet et al. it has been reported that 8% of patients with neurodegenerative disorders were OCB positive [18]. In this study, OCB positivity was also found in 3% of patients with neurovascular disorders [18]. The appearance of OCB after acute cerebrovascular diseases has also been reported by Roström and colleagues. The authors suggested a polyclonal B-cell activation within the CNS after brain tissue damage as the cause [19]. In another study by Prüss and colleagues, an intrathecal immunoglobulin synthesis was even significantly more frequent after stroke compared with controls suggesting an immunologically defined stroke subgroup [20]. An immune mediated mechanism has been suggested in some epilepsy types [21]. While Kowski et al. reported a high incidence of intrathecal immunoglobulin synthesis in patients with so far classified cryptogenic epilepsy (34.1%), Fauser and colleagues detected intrathecal immunoglobulin synthesis only in 8% patients with epilepsy of unknown origin, and in 5% of patients with first seizures of unknown cause, and concluded that the findings were too infrequent and not indicating a leading humoral pathomechanism [22,23].

Altogether, our results are largely in line with previous reports and indicate that OCB can be detected in much lower numbers in non-inflammatory neurological diseases compared to established autoimmune disorders, such as multiple sclerosis (MS), with almost 100% OCB [7]. An intrathecal immunoglobulin synthesis as calculated by Reiber’s diagram was found only in few patients. In general, the determination of OCB is supposed to be more sensitive than Reiber’s diagram in the detection of small amounts of even 0.5% intrathecally synthesized IgG [24].

Discrepancies between the different studies may principally be attributed to the variability of OCB detection methods and to the number of bands chosen as significant in determining the oligoclonal profile [18]. While in most studies immunoblotting was applied after isoelectric focusing in agarose gels, we used polyacrylamide gels with higher resolution and direct detection of OCB by silver staining and assigned 2–3 OCB restricted to CSF as borderline pattern and more than 3 OCB restricted to CSF as distinctively positive. Although the combination of isoelectric focusing/silver staining is demonstrated to be more sensitive in detecting OCB than agarose electrophoresis, there is a lack of data comparing silver staining with immunoblotting [25]. Although the silver staining technique is supposed to be highly sensitive in detection of OCB, immunoblotting might be more specific for IgG due to the antibody-antibody binding reaction [26]. The capillary isoelectric focusing immunoassay (CIEF) represents another promising method, which has been demonstrated as highly specific for the detection of OCBs and even more sensitive compared to current standard methods in a study by Halbgebauer and colleagues [27]. Even though this method is currently not implemented in standard diagnostic procedures, it could be useful when OCBs are highly suspected but tested negative. Additionally, weak OCB positivity with 3% in non-inflammatory controls was also found in this study with CIEF [27].

In recent studies, measuring kappa free light chains, which are bystander products of immunoglobulin synthesis, could be identified as a potential alternative to the current standard method of OCB detection [28,29,30,31]. Interestingly, borderline OCB have been partially confirmed by elevated concentrations of kappa free light chains, a possible surrogate marker for intrathecal immunoglobulin synthesis [28,30,32]. Due to the retrospective nature of our study, data about kappa free light chains are not available for our cohort. We would therefore recommend determining kappa free light chains whenever borderline OCB results are detected, not only when an inflammatory neurological disease is suspected, but also in control groups, to further investigate the potential of this new method.

Another interesting aspect of OCB is, whether they might modify during diseases course. Although OCB are thought to persist in MS, it could be shown that OCB disappeared in 16% of patients who received a second lumbar puncture to exclude multifocal leukoencephalopathy [33]. Since lumbar puncture is an invasive procedure, data about serial CSF diagnostics are scarce in our study, and were only available for patients with CSF diseases, such as normal pressure hydrocephalus and idiopathic intracranial hypertension. However, in all but one OCB positive patients in this group, OCB were also detected in following CSF diagnostics.

It can only be speculated about the origin of intrathecal immunoglobulin synthesis. Since an elevated CSF cell count was used as an exclusion criterion, we support the explanation of other authors of an immunological scar due to a former infection or underlying autoimmune disease [17]. This suggestion might be proven by the application of various tests (Borrelia, syphilis, varicella zoster virus (VZV), herpes simplex virus (HSV), antinuclear antibody (ANA), extractable nuclear antigen (ENA), autoimmune encephalitis, paraneoplastic diseases) [34].

Although routine autoimmune testing was negative in our patients, a contributing immunological pathomechanism might still play a role in non-inflammatory neurological diseases, such as research studies indicated [35]. In neurodegenerative diseases, such as Alzheimer’s diseases, Parkinson’s disease, or amyotrophic lateral sclerosis, increasing evidence point an involvement of immunological cells, which contribute to disease pathogenesis and progression [35,36,37]. Chronic inflammation might then lead to dysregulated immune response that impairs the CNS balance causing dysfunction in large-scale brain networks [38,39]. An involvement of B cells, as detected by intrathecal immunoglobulin synthesis, seems to play a role in cryptogenic epilepsy [23]. However, there is ongoing research attempts to clarify how these CSF antibody interact with neuronal antigens and part of the pathomechanism leading to epileptic seizures [23]. The discovery of anti-N-methyl-D-aspartate (NMDA) receptor encephalitis is an important example, in which antibody could be linked to the occurrence of epileptic seizures [40]. In recent years, several other autoantibodies targeting neuronal structures could be identified [41]. Some of these antibodies can cause movement disorders often with unspecific clinical phenotypes, which can easily be misdiagnosed as neurodegenerative diseases [41]. Patients with an apparently classical non-inflammatory neurological disease and evidence of an intrathecal immunoglobulin synthesis should therefore be of particular interest for further autoimmune research.

Another mechanism may be previous damage to CNS tissue by various insults, which could also elicit a local immune response.

Extended laboratory investigations in our patients largely ruled out previous infections of the CNS or autoimmune processes. However, it also seems possible that even subclinical events can trigger intrathecal immunoglobulin synthesis. For example, neurotrophic viral infections, which could not be detected in routine diagnostic work-up, have been suspected to be the cause of various neurological diseases, including idiopathic cranial nerve neuropathy [42]. Nevertheless, when an immunological scar of a former CNS infection is suspected, determining the pathogen-specific intrathecal antibody synthesis should usually be performed to further clarify the origin. For some pathogens intrathecal antibody synthesis has been observed to persist for years after a CNS infection [1]. Immunological scars to systemic immune reactions (e.g., systemic infections) would also be an explanation for the increasing frequency of mirrored OCB (OCB type 4) with age, suggesting that elderly patients experienced more systemic infections in their life-time. The immune system is supposed to be less effective in the elderly, which is called immune senescence [43]. In particular, the T-cell response against new antigens is reduced [44]. On the other side, T-memory cells may persist up until old age and can be stimulated by vaccination [45]. Antibody profiles reflect therefore rather a growing “fingerprint” of previous antigen contacts than the recent immune status. Furthermore, increasing age is associated with blood-CSF-barrier impairment which may also contribute to a higher percentage of passive diffusion of systemic produced antibodies into the CSF compartment [46].

Our study has some limitations. It is a retrospective investigation of hospitalized patients with neurological diseases. The rate of OCB positivity might therefore be overestimated. However, discrete inflammatory CSF changes may occur even in a small number of healthy patients without neurological diseases. In a group of 99 healthy volunteers, one patient presented 4 OCB and three other patients showed 2 OCB in CSF [47]. Similar proportions of 4% OCB positives in healthy adults have been reported by Haghighi S. et al. [48]. Nevertheless, CSF examination is an important tool in order to exclude autoimmune and in particular infectious diseases. Recently we could show that patients with neurological complications associated with Sjögren’s syndrome did not display a distinct pattern of inflammatory signs in the CSF [49]. However, the amount of OCB positivity was 26% in patients with polyneuropathy and Sjögren’s syndrome, which is clearly higher than in polyneuropathy in the current study. Likewise 8/21 (38%) of patients with Tourette syndrome displayed positive OCB, indicating a possible immune mediated mechanism in the pathogenesis of the disease [50].

## 5. Conclusions

In conclusion, we found OCB in all patients groups with non-inflammatory neurological diseases. Thus, the detection of OCB should not automatically lead to the exclusion of a non-inflammatory neurological disease, but rather point to critically reevaluate clinical characteristics and MRI and laboratory findings. Even an intrathecal synthesis in Reiber’s diagram can be detected occasionally in these patients. Our observations encourage future studies to investigate for an immunological component in the pathomechanisms, especially of those patients who showed an intrathecal immunoglobulin synthesis.

## Figures and Tables

**Table 1:** Immunological findings including intrathecal synthesis of IgM, IgG, and IgA according to Reiber’s diagram and oligoclonal bands restricted to CSF of all groups investigated.

Diagnosis	Patients (*n*)	Female	Age, Mean ± SD (Years)	Intrathecal Synthesis			2–3 CSF Oligoclonal Bands	≥4 CSF Oligoclonal Bands
				IgM	IgG	IgA		
**All patients**	2114	48.1%	52 (±18.8)	0.2%	0.3%	0.0%	3.8%	4.4%
**Symptoms without a neurological disease**	494	60.5%	42 (±17.4)	0.0%	0.2%	0.0%	5.3%	5.5%
Headache	196	60.7%	39 (±17.5)	0.0%	0.0%	0.0%	4.6%	6.6%
Vertigo	43	60.5%	52 (±20.0)	0.0%	0.0%	0.0%	0.0%	4.7%
Paresthesia	138	57.6%	37 (±13.2)	0.0%	0.7%	0.0%	5.1%	3.6%
Pain	117	63.3%	49 (±17.1)	0.0%	0.0%	0.0%	8.6%	6.0%
**Neuropathy**	470	37.9%	58 (±16.1)	0.0%	0.0%	0.0%	2.3%	2.8%
**Peripheral neuropathy**	310	37.4%	62 (±13.3)	0.0%	0.0%	0.0%	1.9%	2.9%
**Cranial nerve impairment**	160	38.8%	50 (±17.8)	0.0%	0.0%	0.0%	3.1%	2.5%
Facial palsy	99	41.4%	44 (±17.3)	0.0%	0.0%	0.0%	5.1%	3.0%
Trigeminal neuralgia	8	62.5%	46 (±13.3)	0.0%	0.0%	0.0%	0.0%	0.0%
Vestibulopathy	12	41.7%	49 (±14.0)	0.0%	0.0%	0.0%	0.0%	0.0%
Oculomotor palsy	41	26.8%	62 (±13.8)	0.0%	0.0%	0.0%	0.0%	2.4%
**Neurovascular diseases**	255	55.3%	60 (±16.3)	0.4%	0.4%	0.0%	4.7%	3.5%
**Epileptic seizure**	264	45.1%	53 (±20.8)	0.0%	0.4%	0.0%	3.8%	4.9%
**Encephalopathy and Delirium**	41	39.0%	67 (±14.4)	0.0%	0.0%	0.0%	4.9%	2.4%
**Muscular diseases**	77	46.8%	50 (±15.7)	0.0%	0.0%	0.0%	1.3%	0.0%
**Neurodegenerativ diseases**	404	40.9%	64 (±14.0)	0.5%	0.7%	0.0%	4.0%	5.5%
Movement disorder	140	42.9%	61 (±16.6)	0.0%	0.0%	0.0%	5.0%	7.1%
Motoneuron disease	147	35.4%	63 (±12.5)	0.7%	1.4%	0.0%	4.8%	3.4%
Dementia	117	44.4%	69 (±10.5)	0.9%	0.9%	0.0%	1.7%	6.0%
**Cerebrospinal fluid flow diseases**	109	58.7%	52 (±19.3)	0.9%	0.9%	0.0%	1.8%	7.3%
Idiopathic intracranial hypertension	59	72.9%	40 (±14.3)	1.7%	1.7%	0.0%	1.7%	10.2%
Normal pressure hydrocephalus	46	39.1%	71 (±9.3)	0.0%	0.0%	0.0%	2.2%	4.4%
Cerebrospinal fluid leakage syndrome	4	75.0%	49 (±15.3)	0.0%	0.0%	0.0%	0.0%	0.0%

**Table 2:** Relative distribution of OCB types for all groups investigated.

Diagnosis	Patients (*n*)	OCB Pattern						
		Typ 1	Typ 2a	Typ 2	Typ 3a	Typ 3	Typ 4	Typ 5
**All patients**	2114	47.7%	2.3%	3.0%	1.5%	1.4%	42.3%	1.8%
**Symptoms without a neurological disease**	494	59.1%	3.2%	4.3%	2.0%	1.2%	29.4%	0.8%
Headache	196	61.7%	3.6%	6.6%	1.0%	0.0%	26.0%	1.0%
Vertigo	43	69.8%	0.0%	0.0%	0.0%	4.6%	25.6%	0.0%
Paresthesia	138	65.2%	2.9%	3.6%	2.2%	0.0%	25.4%	0.7%
Pain	117	43.6%	4.3%	2.6%	4.3%	3.4%	41.0%	0.8%
**Neuropathy**	470	47.2%	1.7%	2.1%	0.6%	0.6%	45.5%	2.1%
**Peripheral neuropathy**	310	43.6%	1.0%	2.3%	1.0%	0.7%	49.4%	2.3%
**Cranial nerve impairment**	160	54.4%	3.1%	1.9%	0.0%	0.6%	38.1%	1.9%
Facial palsy	99	53.5%	5.1%	2.0%	0.0%	1.0%	36.4%	2.0%
Trigeminal neuralgia	8	62.5%	0.0%	0.0%	0.0%	0.0%	37.5%	0.0%
Vestibulopathy	12	66.7%	0.0%	0.0%	0.0%	0.0%	33.3%	0.0%
Oculomotor palsy	41	51.2%	0.0%	2.5%	0.0%	0.0%	43.9%	2.4%
**Neurovascular diseases**	255	38.0%	3.1%	2.0%	1.6%	1.6%	51.0%	2.7%
**Epileptic seizure**	264	45.1%	2.3%	3.0%	1.5%	1.9%	45.5%	0.8%
**Encephalopathy and Delirium**	41	39.0%	2.5%	2.4%	2.4%	0.0%	53.7%	0.0%
**Muscular diseases**	77	46.8%	0.0%	0.0%	1.3%	0.0%	52.0%	0.0%
**Neurodegenerativ diseases**	404	44.8%	1.7%	3.5%	2.2%	2.0%	42.8%	3.0%
Movement disorder	140	44.3%	2.1%	3.6%	2.8%	3.6%	40.0%	3.6%
Motoneuron disease	147	50.3%	2.1%	2.0%	2.7%	1.4%	38.8%	2.7%
Dementia	117	38.5%	0.8%	5.1%	0.8%	0.9%	51.3%	2.6%
**Cerebrospinal fluid flow diseases**	109	41.3%	1.8%	3.6%	0.0%	3.7%	45.9%	3.7%
Idiopathic intracranial hypertension	59	50.8%	1.7%	5.1%	0.0%	5.1%	33.9%	3.4%
Normal pressure hydrocephalus	46	30.4%	2.2%	2.2%	0.0%	2.2%	60.8%	2.2%
Cerebrospinal fluid leakage syndrome	4	25.0%	0.0%	0.0%	0.0%	0.0%	50.0%	25.0%

**Table 3:** Immunological findings including intrathecal synthesis of IgM, IgG, and IgA according to Reiber’s diagram and oligoclonal bands restricted to CSF of patients with neurodegenerative diseases.

Diagnosis	Patients (*n*)	Female	Age, Mean ± SD (Years)	Intrathecal Synthesis			2–3 CSF Oligoclonal Bands	≥4 CSF Oligoclonal Bands
				IgM	IgG	IgA		
Idiopathic Parkinson disease	41	36.6%	66 (±13.1)	0.0%	0.0%	0.0%	7.3%	2.4%
Atypical Parkinson disease	25	40.0%	67 (±9.3)	0.0%	0.0%	0.0%	4.0%	8.0%
Spinocerebellar syndrome	25	60.0%	54 (±15.6)	0.0%	0.0%	0.0%	4.0%	12.0%
Choreatic movement disorder	11	36.4%	51 (±20.9)	0.0%	0.0%	0.0%	18.2%	0.0%
Amyotrophic lateral sclerosis	107	37.4%	64 (±10.5)	0.9%	1.9%	0.0%	6.5%	4.7%
Frontotemporal lobar degeneration	11	27.3%	63 (±4.4)	0.0%	0.0%	0.0%	0.0%	9.1%
Vascular dementia	23	39.1%	74 (±8.8)	0.0%	0.0%	0.0%	4.3%	4.3%

## Data Availability

The data that support the findings of this study are available on request from the corresponding author. The data are not publicly available due to privacy or ethical restrictions.
